# Evaluation of FamMed essentials: a blended-learning program for capacity building of general practitioners in Pakistan

**DOI:** 10.1186/s12909-024-05069-y

**Published:** 2024-03-01

**Authors:** Unab I Khan, Hamida Farazdaq, Azra Naseem, Waseem Suleman, Sania Saleem, Muskaan Abdul Qadir, Komal Fatima

**Affiliations:** 1 Department of Family Medicine, Aga Khan University, Karachi, Pakistan; 2 Blended & Digital Learning Network, Aga Khan University, Karachi, Pakistan; 3 Aga Khan University, Karachi, Pakistan

**Keywords:** Blended learning program, Capacity building in primary care, CIPP framework

## Abstract

**Background:**

To provide access to primary care and universal health coverage, Pakistan requires 60,000 trained family physicians by 2030. At present, most primary care is provided by general practitioners (GPs) who do not have any post-graduate training. Empowering GPs through competency–based programs, that strengthen their knowledge and skills, may be a cost-effective strategy for improving healthcare quality. We describe the development and evaluation of FamMed Essentials, a modular, blended-learning program to improve clinical knowledge and skills of GPs.

**Methods:**

This is a mixed method study. We used the CIPP (content, input, process and product) framework for course development and evaluation. We describe the steps used in content development, strategies for teaching and assessments, and evaluation of strengths and weaknesses of the program. In depth focus group discussions were conducted to gather insight on participants’ and faculty’s perceptions regarding the program’s effectiveness.

**Results:**

Of the 137 participants who have completed the program, 72% were women and 49% had been practicing for more than five years. We saw a significant improvement in knowledge across all modules (*p* = < 0.001) and perceived confidence in clinical skills (*p* = < 0.001). An objective assessment showed participants’ competence in patient management. Participants reported a high level of satisfaction (4.4 ± 0.83 on a 5-point Likert Scale). Focus group discussions revealed a positive impact on clinical practice. Flexibility and use of different teaching and learning strategies were additional strengths. In addition, participants reported an interest in further training. Power outages were highlighted as a major challenge.

**Conclusion:**

In resource-constrained health systems, a modular, blended-learning, competency-based program is helpful to upgrade GPs knowledge without impacting their busy schedules. Accreditation of such programs and provision of a career trajectory for the trained GPs are pivotal to expansion of such initiatives.

## Background

The World Health Organization (WHO) attributes competent Family Physicians (cFP) as a key factor in positively influencing health indicators of a country [1]. Pakistan requires 60,000 cFP by 2030 to improve the primary health system for its population of 220 million [2]. Presently, medical graduates are licensed to practice independently as a general practitioner (GP) after completing a one-year internship without any training in Family Medicine. Family Medicine remains a nascent discipline, and to date, there are only eleven institutions across the country that offer the four-year post-graduate structured training program, after which residents can sit for the FCPS (Fellow of College of Physicians and Surgeons of Pakistan) qualifying exam and be considered a Family Medicine specialist [3]. Moreover, seven institutions across Pakistan offer a two-year structured training, that allows trainees to sit for the MCPS (Member of College of Physicians and Surgeons Pakistan) qualifying examination to be considered a Family Physician [4]. Thus, there aren’t enough programs to train the GP workforce that is required for the country’s healthcare system. As the country moves towards a Family Practice approach to Universal Health Coverage [1], the WHO proposed a one-year diploma in Family Medicine for capacity building of the existing GPs as a cost-effective strategy [5–7]. Recognizing this need [3], nine institutions have created one-year programs that have been endorsed by WHO as Regional Diplomas in Family Medicine [4]. 

The Department of Family Medicine at Aga Khan University developed “FamMed Essentials”, a one-year program, that uses a blended- learning approach to help GPs who are interested in honing their clinical skills.

Experiential and situated learning theories emphasize that learning occurs best when learners participate in authentic activities at the workplace [8]. Technology enhanced learning allows a learning experience with relatively limited resources [9]. However, it is still limited in development of some sub-competencies where physical contact that use human senses are required (e.g., physical examination, diagnostic & therapeutic procedures, etc.). Therefore, a program with a mix/blend of online and onsite learning is a practical solution. Blended learning (BL) is a growing approach for acquiring clinical skills, enabling interaction between participants, facilitators, and resources [10]. It allows continuous learning, while overcoming time and space constraints, and has the potential to improve clinical competencies such as clinical reasoning, history taking, and reflective thinking skills [11]. 

The goal of the program is to help GPs learn and apply core concepts of Family Medicine in their practices. This includes enabling them to provide evidence-based management of common health issues; offer comprehensive care incorporating preventive and health promotive strategies in patients’ health plans; provide contextual care recognizing the role of social determinants of health on patients’ health and wellbeing; and provide timely and appropriate referrals when required.

In this manuscript, we evaluate the effectiveness of the FamMed Essentials program, using the Context, Input Process and Product (CIPP) framework [12], and identify potential areas for improvement to enhance the program’s outcomes related to the needs of GPs in the region.

## Methods

### Study design

This was a mixed-method study utilizing the CIPP framework [13] in the development and evaluation of the program. We chose the CIPP framework as it evaluates the various stages of program development including, understanding the needs/goals, assessing designs/resources, evaluating implementation, and measuring outcomes/impacts. CIPP combines formative and summative assessment, thus aiding implementation and highlighting improvement areas of the program. The contents of the CIPP framework are outlined as below.

#### Context

A context evaluation includes a needs assessment, aligning goals with needs, anticipating barriers and identifying assets and opportunities [13, 14]. Prior to the program a literature search confirmed shortage of competent family physicians [15]. A survey of GPs [16] revealed lack of confidence in managing common outpatient conditions amongst 50% of the respondents. In addition, 62% GPs reported comfort in using technology; and 61% were interested in a flexible, blended-learning program. This evaluation facilitated funding for program development.

#### Input

The process of input and subsequent evaluation addresses whether the targeted needs are achieved by an appropriate plan [14]. Family Medicine faculty met weekly to develop the program’s learning outcomes, objectives, assessments and course content. They reviewed curricula of national and international programs such as Membership of the College of Physicians and Surgeons Pakistan (MCPS); [17] Membership of the Royal College of General Practitioners UK (MRCGP Int) [18]; College of Family Physicians of Canada (CFPC); [19] the Royal Australian College of General Practitioners (RACGP); [20] American Academy of Family Physicians (AAFP); [21] and World Health Organization (WHO) [22]. Topics were evaluated based on national burden of disease [1, 23]. The Delphi method was used to reach consensus on the final topic list [24]. 

The program content was organized into five modules: Pediatric and Adolescent Health, Reproductive Health, Non-Communicable Diseases (NCDs), Common infections and Health of special populations (palliative care, and geriatrics), and Beyond infections and NCDs (including common musculoskeletal, gastroenterological, and surgical problems). Faculty were trained in online and blending learning methods. The multimedia development team created engaging graphical voice-overpower Point presentations. The program was hosted on a virtual learning environment (VLE) platform. It was evaluated by the Department of Continuing Professional Education (DCPE) and accredited for 310 Accreditation Council of Continuing Medical Education (ACCME) credits. It was also endorsed by WHO-EMRO as a Regional Diploma in Family Medicine.

#### Process

The process describes if the program was effectively implemented and evaluates its strengths and weaknesses which help with further refinement of a program [25].

FamMed Essentials is promoted through university communication channels and social media platforms (Facebook page, Twitter, Linked in) and various WhatsApp groups.

Participants feedback is collected through a questionnaire to refine the program based on strengths and weaknesses.

#### Product

The product evaluation component in CIPP closely resembles a traditional “summative” program evaluation. It assesses the program outcomes, both positive and negative [12]. Table 1 shows the components used for evaluation of each element of the framework.

**Table 1:** Components in evaluation of each element of CIPP framework

Type of Evaluation	Questions asked	Methods
Context	Is there a need to develop a program?	- Literature review
	What are the educational needs of general practitioners?	- Needs assessment survey conducted
	What resources are required for content development, administration of program and funding?	- Identification of existing resources - Family Medicine Faculty - Funding from University
Input	How appropriate is the content for an LMIC*, especially Pakistan? Does it cover the core material of national and international programs?	- Review of curricula of existing Family Medicine post-graduate training programs - Consensus of faculty using Delphi method
	What eeducational strategies are used for optimal content delivery?	- Training of faculty by Blended and Digital Learning network team - Use of synchronous, asynchronous, and face-to-face components - Multimedia team
	How user friendly is the program in the virtual learning environment?	- Learning management system team (LMS)
	What is the accreditation process for continuing professional development?	- Department of Continuous Professional Education evaluates for ACCME credit hours
	How will the program be most accessible to GPs?	- Modular curriculum - Curricular content available for asynchronous learning - Synchronous sessions scheduled on weekends
Process	Are we inducting the right participants?	- Broad reach through University’s online recruitment platforms - Demographics of participants
	To what extent the program was carried out as planned?	- Participants’ feedback at the end of each module, onsite clinical skills session and Task Oriented Assessment of Clinical Skills (TOACS).
	Program adherence and program attrition	- Ongoing monitoring of participation and completion of activities. - Reminders sent to inactive participants.
Product	To what extent did the program achieve the original objectives?	- Knowledge acquisition through pre and post module tests - Perceived confidence in clinical skills through feedback after onsite clinical skills session - Competence in knowledge and clinical skills through TOACS exam.
	What is the ooverall impression and impact of the program? Is the program sustainable?	- End of program survey - Participant Focus Group Discussion (FGDs) - FGD with the Faculty - Financial feasibility work plan

*Low and Low Middle-Income Countries

### Program participants and eligibility

The program has been specifically designed for GPs practicing in Pakistan who hold a valid license to practice. In addition, physicians trained in Pakistan, but residing abroad also showed interest in improving their clinical knowledge and skills and were included. Furthermore, the University is a strong proponent of empowering nurses, therefore, we also welcomed nursing students pursuing a master’s degree in the clinical stream and interested in becoming nurse practitioners into the program.

### Setting

FamMed Essentials utilizes a modular and blended learning approach, allowing participants to access the program content through the Aga Khan University (AKU) virtual learning Environment (VLE). The onsite component of the program is conducted at the state-of-the-art Center for Innovation in Medical Education (CIME) at the Aga Khan University in Karachi, Pakistan.

### Educational/teaching intervention

**Fig. 1:**
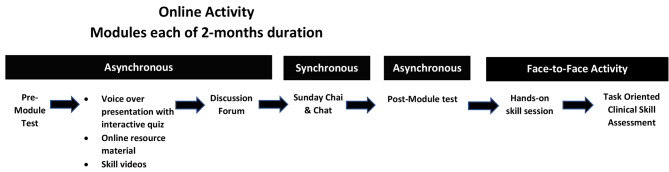
Overview of FamMed essentials blending learning program

FamMed Essentials is a flexible one-year modular program designed for busy practitioners. The overview of the program and the structure of each module can be seen in Fig. 1.

Each module begins with a mandatory pre-module test consisting of multiple-choice questions (MCQs) to assess prior knowledge and identify knowledge gaps. The module content includes videos covering 15–16 topics, featuring text, figures, algorithms and narrative audios from Family Medicine faculty. These videos also include embedded quizzes to promote learner’s engagement [26]. Additional resources and clinical skills videos are also available (asynchronous independent activity). Learners can post their questions on a visual board using a Padlet® wall, which automatically sends an email to the module coordinator. Asynchronous collaborative activities include a monthly discussion forum. Monthly synchronous sessions called “Sunday Chai and Chat” (tea in local language) with faculty provide an opportunity to discuss these questions, reinforce key concepts through interactive quizzes, role plays and engage in small-group case-based discussions in breakout rooms. At the end of each module participants must complete a mandatory post-module test before accessing the next module. After completing five modules, participants attend a three-day onsite clinical skills session, to learn essential skills on simulators and simulated patients (e.g. breast and pelvic examinations, laceration repairs and musculoskeletal examinations).

This is followed by a 14-station Task Oriented Assessment of Clinical skills (TOACS) exam with feedback and sign off. It assesses history taking, physical examination, diagnosis management and/or counselling for outpatient conditions. Evaluation is conducted using a standardized marking grid.

### Data collection tools and statistical analysis

We collected data for various parameters in the program using the following instruments:

- Improvement in knowledge was assessed through comparing the scores of pre- and post- module tests obtained from the VLE using paired t-test. A *p*-value of < 0.005 was considered statistically significant. We used STATA v. 16.0 for quantitative analysis.
- Level of confidence in clinical skills were evaluated through a feedback form completed by participants at the conclusion of the onsite clinical skills session.
- Clinical management skills were assessed objectively through a TOACS exam.
- All participants who completed the program were invited to fill in an online survey using Survey Monkey®. The survey included questions regarding the acceptability and perceived utility of each component of the program.
- To obtain an in-depth understanding of participants’ experiences, four focus group discussions (FGDs) were conducted with 12–15 participants in each group. Each FGD followed a question script and lasted 45–60 min. A separate FGD was conducted with the faculty who helped develop and run the program. FGDs were audio recorded and transcribed and checked for accuracy. Two members of the evaluation team (HF and SS) decoded and sorted content by themes. To develop consensus three members (HF, SS and UIK) crossmatched the themes.
- Sustainability evaluation was based on the operational budgetary requirements.

#### Ethics approval

We received approval from the University Ethics Review Committee (ERC: 2021-6431-2013) for conducting the evaluation. We obtained informed consent from all participants in the study during the focus-group discussions and whilst completing the online survey.

## Results

### Participants’ demographics

Five cohorts have completed FamMed Essentials. The initial three groups accommodated 20 participants each, with later cohorts expanding due to increased faculty confidence. During this time, 214 participants registered in the program and 28 (13%) formally withdrew. Of the 186 participants, 137 (74%) completed the program and 49 (26%) are at various stages of completion. Thus, this evaluation focuses on the 137 participants who have completed the program.

The majority of participants were women (n:99, 72%), reflecting the trend in Pakistani medical school admissions [27]. While the majority were from Karachi (n:94, 69%), participations also came from rural areas (n:11, 8%), and other urban cities (n:20, 14%). In addition, few participants were residing in the Middle East (n:8, 6%) and Canada (n:4, 3%). About half (n:67, 48%) were in practice for ≥ 5 years; (n:62, 45%) for < 5 years; and 8 (6%) had never practiced. A majority (n:102, 75%) were practicing in different settings including hospitals (n: 53, 39%); private clinics (n:15, 11%); and government clinics (n: 8, 6%). In addition, 26 (19%) were working in non-clinical settings; and 35 (25%) were unemployed.

## Program outcomes

### Quantitative analysis

#### Improvement in clinical knowledge

There was significant improvement in clinical knowledge in all modules, with the largest increase in scores noted in women’s health and management of non-communicable diseases (Table 2).

**Table 2:** Knowledge Change and Clinical Skills Confidence Assessment

**Knowledge Change**			
**Module**	**Pre-test scores** **mean ± SD (range)**	**Post-test scores** **mean ± SD (range)**	***p*** -value*
Paediatric and Adolescent Medicine	22.8 ± 6.2 (1.5–37)	37.1 ± 5.4 (23.5–45)	< 0.001
Reproductive and Genitourinary concerns	15.8 ± 5.2 (0–28)	32.1 ± 5.2 (13–40)	< 0.001
Non-communicable Diseases	16.3 ± 5.0 (0 − 28.3)	34.2 ± 5.2 (20–41)	< 0.001
Infectious diseases & Health of Special Populations (geriatric &palliative care)	21.1 ± 5.6 (0–31)	36.8 ± 5.1 (26–43)	< 0.001
Beyond Infections (musculoskeletal, surgical, and gastroenterological conditions)	22.1 ± 7.4 (0–40)	36.9 ± 5.5 (20–45)	< 0.001
Confidence** in Performing Clinical Skills			
**Clinical Skills**	**Pre-session**	**Post -session**	***p*** **-value**
Ear examination (otoscopy)	2.3 ± 0.1	3.9 ± 0.1	< 0.001
Eye examination (fundoscopy)	1.7 ± 0.1	3.9 ± 0.1	< 0.001
Neck and thyroid examination	2.7 ± 0.1	4.4 ± 0.1	< 0.001
Use of peak flow meter and inhaler	2.6 ± 0.2	4.3 ± 0.1	< 0.001
Assess patient with chest pain	3.0 ± 0.1	4.5 ± 0.1	< 0.001
Breast examination	2.9 ± 0.1	4.7 ± 0.1	< 0.001
Vaginal examination	2.3 ± 0.2	4.3 ± 0.1	< 0.001
Shoulder examination	2.0 ± 0.1	4.1 ± 0.1	< 0.001
Knee examination	2.3 ± 0.1	4.3 ± 0.1	< 0.001
Foot examination for diabetes	2.5 ± 0.1	4.6 ± 0.1	< 0.001
Local anaesthesia	2.5 ± 0.2	4.4 ± 0.1	< 0.001
Management of abdominal emergencies	2.5 ± 0.2	4.2 ± 0.1	< 0.001

*: paired t-test

**: 5-point Likert scale: 1 = not confident at all; 2 = somewhat confident; 3 = neutral; 4 = somewhat confident; 5 = very confident

#### Improvement in clinical skills

There was a significant improvement in participants’ perceived confidence in performing clinical skills taught during the onsite clinical skills session (Table 2).

#### Competence in management of common clinical issues

All participants completed a Task Oriented Assessment of Clinical Skills exam. Table 3 shows the average score and the pass rate at each station. The list of stations is longer than 14 as some stations were changed for each cohort. While most participants performed well on management of NCDs, they scored lower in management of pediatric conditions such as malnutrition. Participants improved in managing NCDs as they encounter more adult cases in their practices [16]. We recognize the limited pediatric teaching available at government and private medical institutions and are working on enhancing exposure to pediatric cases within the program.

**Table 3:** Results of Task Oriented Assessment of Clinical Skills

Station	Number of participants	Score* (mean ± SD)	Pass N (%)
Diabetes	76	3.3 ± 0.78	64 (84)
Gastritis	76	3.3 ± 0.97	62 (82%)
UTI	112	3.5 ± 0.79	101 (90)
Menorrhagia	76	3.5 ± 0.68	68 (90)
Knee pain	107	3.6 ± 0.83	96 (90)
Enteric Fever	77	3.0 ± 1.4	53 (69)
Depression	112	3.6 ± 0.63	106 (95)
Headache	46	3.4 ± 0.57	44 (96)
Diarrhea	122	3.5 ± 0.83	111 (91)
Breaking bad news	137	3.0 ± 1.2	103 (75)
Acute chest pain	122	3.1 ± 1.3	84 (69)
Acne	52	3.0 ± 0.92	38 (73)
Dengue fever	60	3 ± 1.06	43 (72)
HTN	45	3.67 ± 0.56	43 (96)
Red eye	15	3.5 ± 0.64	14 (93)
Asthma	30	3.5 ± 0.54	26 (87)
Child with malnutrition	15	2.5 ± 0.99	8 (53)
Acute appendicitis	15	3.33 ± 0.62	14 (93)
NCD management	36	3.58 ± 0.55	35 (97)
PCOS	61	3.31 ± 1.16	49 (80)

*: 1 = Clear Fail, 2 = Borderline Fail, 3 = Borderline pass, 4 = Clear Pass

#### End of Program survey

Ninety-five participants completed the online survey evaluating all program components (Table 4). Overall, the participants reported high satisfaction with most components. The lowest scores were for the discussion fora (4.1 ± 0.9 on a 5-point Likert scale). Participants who were in clinical practice had higher satisfaction with components requiring application of clinical knowledge.

**Table 4:** Participants’ satisfaction with program and its components

	Total Score*	Not in clinical practice	In clinical practice	*p*-value
Overall Satisfaction	4.4 ± 0.83	4.4 ± 0.2	4.4 ± 0.1	0.913
Knowledge gained from program	4.6 ± 0.71	4.7 ± 0.1	4.6 ± 0.1	0.658
Skills learned from program	4.6 ± 0.61	4.4 ± 0.1	4.6 ± 0.1	0.168
Engagement during program	4.3 ± 0.8	4 ± 1.1	4.3 ± 0.7	0.109
Interaction with faculty	4.5 ± 0.5	4.3 ± 0.2	4.5 ± 0.1	0.164
Help create patient care plans	4.3 ± 0.7	4.2 ± 0.5	4.3 ± 0.1	0.332
Clinical skills session	4.6 ± 0.6	4.3 ± 0.2	4.7 ± 0.1	0.017
Sunday chai and chat sessions	4.6 ± 0.7	4.2 ± 0.2	4.5 ± 0.1	0.043
Discussion fora	4.1 ± 0.9	3.7 ± 0.3	4.2 ± 0.1	0.039
TOACS**	4.5 ± 0.6	4.3 ± 0.2	4.6 ± 0.1	0.043

*: 5-point Likert scale with 1 = Very Unsatisfactory, 2 = Unsatisfactory, 3 = Neutral, 4 = Satisfactory, 5 = Very Satisfactory

**: Task oriented assessment of clinical skills

### Qualitative analysis

The analysis of participants’ FGDs yielded four main themes that are presented in Table 5. The focus group with faculty revealed both positive opinions and challenges. Most faculty felt that developing content and skills’ sessions helped update their own knowledge and skills. In addition, they recognized their own professional development through the training in blended teaching and learning skills, that they are now using in other classes. Faculty felt that the diverse learning needs of the participants were addressed by using multiple teaching and learning strategies (e.g. role plays, discussions, and use of independent learning resources). All faculty wanted to continue being part of FamMed Essentials.

**Table 5:** Participants’ perceptions and relevant quotes related to themes

Themes	Quotes
Effectiveness of online modality	• “Flexibility of the program’s online platform and the convenience of revisiting the content.” • “It was a good opportunity for updating my knowledge and simultaneously take care of my household responsibilities.” • “Internet disruptions as the main hindrance” • “Program’s compatibility with mobile phones needs to be improved.”
Effectiveness of different teaching strategies:	• Power point presentations were relevant and concise ensuring attention, retention, and easy absorption.” • “Case-based discussions during the Sunday Chai and Chat sessions were most helpful.” • “Splitting in small groups in break out rooms provided opportunity for active participation and interaction with faculty and other participants.” • “Onsite clinical skill session and skill videos was beneficial for revising examination skills.” • “It was worth travelling from Canada for the onsite clinical skill sessions.” • “The discussion fora were least helpful component. Repetition in individual responses made the activity boring. • “Engaging with discussion forum threads motivated me to independently research the topic, contributing significantly to my knowledge.” • “Not everyone is active in discussion forum, moreover people have different capabilities.” • “Due to time zone differences by the time I open, a lot has already happened in the forum, hence it is difficult for some to participate in the assigned time.” • “TOACS, it taught me how to interact with patients in a real setting.”
Utilization of knowledge and skills:	• “It wasn’t just clinical knowledge but the improvement in systematic approach to data gathering and diagnosis that was actually the strength of the program.” • “It has made me more confident in data gathering and to arrive at a diagnosis.” • “We used to simply write prescriptions based on symptoms. Now we learned about data gathering and arriving at a possible diagnosis.” • “Counselling and communication skills learnt will enable in addressing most patient problems while working in communities.” • “I have seen a rise in number of both new and follow up patients at my private practice clinic.”
Help with professional development:	• “The course has stimulated my interest in professional growth, and I plan for post-graduate certification exams (MRCGP int or MCPS in Family Medicine).” • “I was stuck after graduation and internship; the program has provided direction to move forward in my professional journey.” • “I am planning for MRCGP (int) exam after a career break of 4 years.” • “I now feel confident to take up qualifying exam in Family Medicine which will pave my way for practicing in Canada for the time I am here.” • “I feel confident to pursue becoming a nurse practitioner.”

### Program sustainability

The program received support from the University Provost and Dean of the Medical College for development. Pfizer pharmaceuticals provided an educational grant for scholarship for 23 participants.

The program is currently offered twice a year with up to 100 participants per cohort, covering cost of faculty time and administrative needs. There is interest from other low-and-middle income countries (LMICs) to expand the program.

## Discussion

Our evaluation findings demonstrate that FamMed Essentials is a sustainable and adaptable capacity building model for GPs in Pakistan and the region. Both quantitative and qualitative data indicate that the blended learning approach enhances clinical knowledge, clinical skills, and self-reflection. Participants also report improved patient-centeredness and communication skills. The program is valued for professional growth with participants intending to sit for qualifying examinations. FamMed Essentials fills a crucial gap in resource -constrained health systems by increasing competence in managing common outpatient conditions. Other programs in the country and the region have reported strengths and challenges. The one-year diploma offered through Khyber Medical University reports enhanced evidence-based practice in participants; and identified time constraints and technology availability as barriers [28]. Similarly, participants at a two-year, in-person diploma offered through Ziauddin University (a private institution) report improvement in problem solving skills, but identify taking time off from work as a major challenge [29]. The 12- month regional diploma offered by the American University of Beirut reports improvement in clinical skills of general practitioners. The authors identified accreditation/recognition by local regulatory bodies and career integration as a major challenge [30]. An evaluation of the diploma in Saudi Arabia reported development of proficient consultation skills and patient-centered care among the participants [31]. Other countries such as Iran have used short diplomas to strengthen the primary health care infrastructure and bridge health care delivery gaps [32]. 

Several challenges were identified by participants of FamMed Essentials, including frequent internet disruptions, which are common in LMICs. Other studies have recognized that connectivity issues hinder learning and the need to address this to enhance learning experience [33]. The flexibility of our program allows access that supports learning. Participants reported less confidence in managing pediatric cases due to insufficient pediatric exposure during their medical education. To address this, we have integrated additional child health topics and skills into the curriculum. We also noted that participants in clinical practice reported a higher satisfaction with components requiring application of clinical knowledge. A clinical attachment program may help aspiring practitioners in enhancing their skills.

Our study found real-time discussions with experts, videos and skills development sessions were the most effective methods. Other studies highlight the importance of interactions between learners and teachers in online courses [34–36]. The challenge noted by our participants regarding the use of discussion forums is also consistent with existing research [37]. There is a need to address the readiness of both faculty and students to engage in asynchronous discussions when designing online courses.

As the program is new, we have not yet observed participants’ career trajectory. In addition, we were unable to observe pre- and post- program patient care in the physicians’ workplace. This is an important area to ensure the application of knowledge and skills in clinical practice.

## Conclusion

The evaluation of FamMed Essentials demonstrates the effectiveness of blended learning in enhancing GPs’ clinical skills in resource limited settings, thereby enhancing overall primary care. Accreditation of these programs and government support in providing a career trajectory to these trained GPs are pivotal to expansion of such initiatives. Lastly the CIPP framework proved crucial for this assessment and is recommended for similar health care programs aiming to adopt innovative teaching.

## Data Availability

These are available from the corresponding author on reasonable request.

## Abbreviations

CIPP Framework: Content, Input, Process and Product
GPs: General Practitioners
WHO: World Health Organization
cFP: Competent Family Physicians
VLE: Virtual Learning Environment
MCQ: Multiple Choice Question
TOACS: Task Oriented Assessment of Clinical Skills
MCPS: Membership of College of Physicians and Surgeons Pakistan
MRCGP: Membership of the Royal College of General Practitioners
CFPC: College of Family Physicians of Canada
RACGP: Royal Australian College of General Practitioners
AAFP: American Academy of Family Physicians
NCD: Non-Communicable Diseases
DCPE: Department of Continuing Professional Education
ACCME: Accreditation Council of Continuing Medical Education
FGD: Focus Group Discussion
LMIC: Low-and-Middle Income Countries

## References

[1] Organization WH. Conceptual and strategic approach to family practice: towards universal health coverage through family practice in the Eastern Mediterranean Region. 2014.

[2] Alwan A (2016). Overview of the 63rd session of the WHO Regional Committee for the Eastern Mediterranean. East Mediterr Health J.

[3] Andrades M (2019). Family Medicine: indispensable for an effective health care system. Annals of Jinnah Sindh Medical University.

[4] Tejani FA, Rashid MA. Enhancing family medicine training to build capacity in Pakistan: a call for action. Educ Prim Care. 2023:1–4. 10.1080/14739879.2023.2204340 37159548

[5] Sanaiey NZ, Karamnejad S, Rezaee R (2015). Educational needs of family physicians in the domains of health and conformity with continuing education in Fasa University of Medical Sciences. J Adv Med Educ Professionalism.

[6] Zink T, Solberg E (2014). Development of a global health curriculum for family medicine based on ACGME competencies. Teach Learn Med.

[7] Arya N, Dahlman B, Gibson C, Ponka D, Haq C, Rouleau K (2017). Developing family practice to respond to global health challenges: the Besrour papers: a series on the state of family medicine in the world. Can Fam Physician.

[8] Yardley S, Teunissen PW, Dornan T (2012). Experiential learning: AMEE guide No. 63. Med Teach.

[9] Nicoll P, MacRury S, Van Woerden HC, Smyth K (2018). Evaluation of technology-enhanced learning programs for health care professionals: systematic review. J Med Internet Res.

[10] Salim H, Lee PY, Ghazali SS, Ching SM, Ali H, Shamsuddin NH (2018). Perceptions toward a pilot project on blended learning in Malaysian family medicine postgraduate training: a qualitative study. BMC Med Educ.

[11] Rowe M, Frantz J, Bozalek V (2012). The role of blended learning in the clinical education of healthcare students: a systematic review. Med Teach.

[12] Stufflebeam DL. CIPP evaluation model checklist: a tool for applying the CIPP model to assess projects and programs. Western Michigan University Evaluation Center; 2015.

[13] Burke E, Hennessy M (2021). Evaluation of an early career clinical academic training programme using the CIPP model. BMJ open.

[14] Toosi M, Modarres M, Amini M, Geranmayeh M, Context. Input, process, and product evaluation model in medical education: a systematic review. J Educ Health Promotion. 2021;10(1). 10.4103/jehp.jehp_1115_20 PMC8249974 34250133

[15] Sabzwari SR (2015). The case for family medicine in Pakistan. J Pakistan Med Association.

[16] Farazdaq H, Gilani JA, Qureshi A, Khan UI (2022). Needs assessment of general practitioners in Pakistan: a descriptive cross-sectional survey. J Family Med Prim Care.

[17] MCPS Family Medicine Curriculum [cited 2023 Feb 6]. Available from: https://listing.cpsp.edu.pk/weblink_new/training_program/mcps.php.

[18] MRCGP [INT] Curriculum: https://mrcgpintsouthasia.org/wp-content/uploads/2023/06/Exam-Curriculum.pdf.

[19] Triple C. Competency Based Curriculum [cited 2023 Feb 6]. Available from: https://www.cfpc.ca/en/education-professional-development/educational-frameworks-and-reference-guides/triple-c-competency-based-curriculum.

[20] RACGP curriculum and syllabus for Australian general practice [cited. 2023 Feb 6]. Available from: https://www.racgp.org.au/education/education-providers/curriculum/curriculum-and-syllabus/home.

[21] AAFP. Family Medicine Residency Curriculum Guidelines [cited 2023 Feb 6]. Available from: https://www.aafp.org/students-residents/residency-program-directors/curriculum-guidelines.html.

[22] WHO Eastern Meditteranean Region [cited 2023 Feb 6]. Available from: https://www.emro.who.int/health-topics/primary-health-care/.

[23] Redwood-Campbell L, Pakes B, Rouleau K, MacDonald CJ, Arya N, Purkey E (2011). Developing a curriculum framework for global health in family medicine: emerging principles, competencies, and educational approaches. BMC Med Educ.

[24] Ab Latif R, Dahlan A, Mulud ZA, Nor MZM. The Delphi technique as a method to obtain consensus in health care education research. Educ Med J. 2017;9(3).

[25] Frye AW, Hemmer PA (2012). Program evaluation models and related theories: AMEE guide no. 67. Med Teach.

[26] Martin F, Borup J (2022). Online learner engagement: conceptual definitions, research themes, and supportive practices. Educational Psychol.

[27] Baig LA (2020). Women empowerment or feminism: facts and myths about feminization of medical education. Pakistan J Med Sci.

[28] Khan AJ, Sethi A, Fazid S, Haq ZU, Raza J, Patel M (2022). How does postgraduate diploma in Family Medicine impact on primary care doctors?. BMC Med Educ.

[29] Ali R, Farooqui A, Arshia S, Naqvi A. Evaluation of diploma in family medicine ensuring quality through CIPP model.

[30] Salah H, Mataria A, Wajid G, Mandil A, Hamadeh G, Osman M (2021). Promoting family practice-based model of care: the role of WHO’s professional diploma in family medicine in the Eastern Mediterranean Region. East Mediterr Health J.

[31] Al-Khathami AD (2012). Evaluation of Saudi family medicine training program: the application of CIPP evaluation format. Med Teach.

[32] Dargahi H, Darrudi A, Zalvand R (2019). Family medicine program in Iran: SWOT analysis and tows matrix model. Iran J Public Health.

[33] Le N (2022). Literature Review on the barriers to Online Learning during Covid-19 pandemic. Open Access Library Journal.

[34] Blaine AM (2019). Interaction and presence in the virtual classroom: an analysis of the perceptions of students and teachers in online and blended Advanced Placement courses. Comput Educ.

[35] Vlachopoulos D, Makri A (2019). Online communication and interaction in distance higher education: a framework study of good practice. Int Rev Educ.

[36] Saiyad S, Virk A, Mahajan R, Singh T (2020). Online teaching in medical training: establishing good online teaching practices from cumulative experience. Int J Appl Basic Med Res.

[37] Naseem A, Nizamuddin S, Ghias K (2022). The outcomes of a mobile just-in-time-learning intervention for teaching bioethics in Pakistan. BMC Med Educ.

